# On Whether Sex Is Binary

**DOI:** 10.1007/s10508-025-03400-2

**Published:** 2026-01-04

**Authors:** Brian D. Earp, Morgan Carpenter, Sebastian Porsdam Mann

**Affiliations:** 1 Centre for Biomedical Ethics, Yong Loo Lin School of Medicine, National University of Singapore, Queenstown, Singapore; 2 Sydney Health Ethics, School of Public Health, The University of Sydney, Sydney, Australia; 3 Center for Advanced Studies in Bioscience Innovation Law (CeBIL), Faculty of Law, University of Copenhagen, Copenhagen, Denmark

The call for commentaries asks, “How many sexes are there? How many genders are there?” In what follows, we attend to the first of these questions. We argue that disagreements about how many sexes there are often stem from people using the word “sex” to pick out different things in different contexts—sometimes an abstract evolutionary model based on idealized body plans (interpreted functionally in terms of hypothetical gamete production), and sometimes, the diverse ways that actual human bodies are organized in practice. Clarifying which sense is in play, and why, is essential to avoid talking past one another. We introduce an analogy—through a “Toy Factory” thought experiment—to help illustrate our argument.

We’ll start with some clarifications. First, the wording of the question (“How many sexes are there?”) presupposes that sexes are discrete countable kinds, which may itself be a point of contention in some discourses. For example, some might argue that sex is best understood as a spectrum (e.g., Fuentes, 2025), a multidimensional phenomenon (e.g., Fausto-Sterling, 2000), a context-sensitive pragmatic concept (e.g., Richardson, 2013, 2022), or something similar. On such views, the question “How many?” may be ill-posed from the outset. Nevertheless, we will proceed with what we take to be the spirit of the question, which seems amenable to different interpretations.

One way to interpret the question is as a shorthand for asking whether sex is binary. This claim, too, can be interpreted in various ways. Some of these interpretations might seem compatible with views according to which sex is (also) a spectrumlike or multidimensional phenomenon. For example, the purported spectrum could be described as being anchored at two different poles; the posited multidimensional object could be said to consist of two main feature clusters (e.g., Hodson et al., 2019), and so on. This sense of “binary” implies the existence of two main prototypes with overlapping features and fuzzy boundaries. Other interpretations of “binary” are stricter. For example, one might be asking whether there are two—and only two—exhaustive and mutually exclusive sex categories, male and female, that apply universally. On such a view, sex itself (as opposed to, say, various sex-related attributes, or methods of determining sex) would not be multidimensional or a spectrum, and nor would it have fuzzy boundaries (e.g., Byrne, 2024; Coleman, 2024; Wright, 2025).

This latter formulation is sometimes taken to bear on the question of how people with intersex traits or differences of sex development (DSDs) should be categorized, given that some such individuals may not be straightforwardly classifiable in terms of such (strictly interpreted) binary categories, or, alternatively, can reasonably be classified in multiple ways with respect to such categories (Liao, 2022; Reis, 2021). It is important to note, however, that people with DSDs do not tend to regard themselves as belonging to a “third” sex category, either (though there may be some exceptions to this; see Morland, 2016). Suffice to say, there is debate and disagreement about the matter even within intersex communities (Carpenter, 2018).

Intersex advocacy organizations, for their part, tend to support pluralism and personal preference in relation to sex self-classification among people with DSDs, emphasizing that their main concern is to resist the needless pathologization of physically benign or low-risk bodily variations, particularly when this leads to non-consensual genital or reproductive interventions (for a recent international overview of such concerns, see the Brussels Collaboration on Bodily Integrity, 2025).

Finally, contemporary disputes about whether sex is binary, or how many sexes there are, typically arise in the context of broader (often quite acrimonious) debates about gender and/or gender identity, in which intersex people are sometimes invoked in ways that instrumentalize their existence. This happens, for instance, when people with DSDs are primarily mentioned in the context of various political or ideological skirmishes in the so-called “gender wars.” To avoid such potential instrumentalization, in what follows we will restrict our focus to the question of sex, leaving questions about gender mostly to one side. However, in doing so, we do not imply that such questions are unimportant or irrelevant, nor even that they can be cleanly separated. For example, a person's subjectively experienced gender may be caused, in part, by sex-linked biological factors; and how a person is classified in terms of sex—particularly in cases of perceived ambiguity—may be influenced by societal gender norms (see Reis, 2021). Instead, we seek only to make the discussion more manageable, which requires that we adopt a narrower focus.

## One Interpretation: Sex in Theory

One thing the word “sex” has been used to pick out is a theoretical model developed by evolutionary biologists to predict and explain various features of the natural world. This model includes a normative or idealized “design plan” for bodies in (anisogamous) sexually reproducing species such as ours. The design plan for bodies in a given species—what we’ll call its “body plan” going forward—refers in this context to the evolutionarily maintained pattern of traits that enables species members to reproduce. This pattern results from natural selection. Anisogamous species are, by definition, those that reproduce through the fusion of two very differently sized gametes. Anisogamous means “unequal union.” This method of reproduction is employed by virtually all animals, including humans, as well as most plants (Togashi & Cox, 2011).

According to this sex-as-model approach, members of anisogamous species can usually be parsimoniously divided into groups based on their approximate adherence to one of two idealized body plans. Bodies that closely approximate the first plan are functionally and structurally organized in a way that usually leads to the production of large gametes (ova or eggs), whereas those that closely approximate the second plan are functionally and structurally organized in a way that usually leads to the production of small gametes (sperm).

So, if by “sex” one means “the abstract distinction between evolutionarily maintained body plans in anisogamous species, according to which type of gamete each idealized body plan is structurally and functionally organized to usually produce,” the answer to how many sexes there are is “two.” This is because—as proponents of this approach are wont to emphasize—there are exactly two types of gametes in anisogamous species like ours: large and small, with no intermediates.^1^ Since each idealized body plan has been “designed” by evolution to produce just one of these two types of gametes (and there are no other types of gametes in anisogamous species), there are two such idealized body plans, neither more nor less.

So, sex is binary.

That is one view. But it faces certain limitations. One is that idealized—as opposed to actual—body plans for hypothetical gamete production do not, strictly speaking, exist. They are, instead, theoretical constructs or abstractions: simplified models that purport to explain sex as it is found in various species, but which are not identical to sex itself (this is roughly analogous to the way that the blueprint for a building is not identical to the building). Moreover, even when considered as models, although they are indeed predictive of many sex-related phenomena with a high degree of accuracy, they do not fully account for the observable diversity of sex as it exists in the real world. Thus, when we move from (simplified) model to (complex) reality, we find that actual body plans in many anisogamous species are variable, differing along numerous dimensions (albeit, with certain structural and functional similarities in the vast majority of cases) (Liao, 2022). These bodies, in turn, can in principle be classified according to various criteria, with real or hypothetical gamete production (among other criteria of interest to evolutionary biologists) being just one of them (Meynell, 2024).

Can we nevertheless rescue the binary understanding of sex in its strictest sense—the sense according to which male and female are exhaustive and mutually exclusive sex categories admitting of no exceptions? It is true that there are only two gamete types in anisogamous species such as ours, and that these types are mutually exclusive and exhaustive (see the footnote for details). However, the question “How many gamete types are there?” is not the same as the question, “How many sexes are there?” and it is the latter question we are supposed to be answering.

## Asking Questions

But why was this question posed in the first place? This meta-question, we suggest, might provide a clue as to what sort of answer (or answers) to “How many sexes are there?” would be fitting or make sense. After all, different people might have different aims or purposes in asking what sex is, or how many sexes there are. Accordingly, they might end up using the same word “sex” to mean different things in different contexts. So, to avoid talking past one another, it is important to be clear about which use of “sex” is operative in which context and why.

In our view, it can sometimes be appropriate to use “sex” in a way that differs from the idealized-body-plan approach described previously. One reason for this is that some human persons might reasonably wish to classify themselves, or perhaps others, in terms of sex. And yet persons are not, nor can they be reduced to, abstract or idealized body plans (or approximations thereof). Rather, each individual person has a particular body with its own specific set of features. These features, along with the developmental pathways that produced them, may not always line up with the abstractions modeled by evolutionary biologists.

The design plan is not the manifested reality. And yet, the question, “How many sexes are there?”—in relation to humans, at least—almost only ever arises, historically speaking, in connection with certain background political debates about the appropriate classification of actual persons (the manifested reality), not idealized body plans. Thus, if one argues, in response to such debates, that the very concept of sex necessarily picks out the distinction between idealized body plans based on hypothetical gamete production (rather than, say, a distinction between different ways that actual people may be embodied), one is using the term “sex” in a way that departs from the sense implied by the debates in question. In other words, one is talking past one’s interlocutors.

Of course, one could stipulate that the latter, debate-relevant sense of “sex” is simply mistaken. In other words, one could argue that “sex” as it is invoked in contemporary sociopolitical debates refers not to sex itself, but rather, to ways that sex is manifested or determined (i.e., to “downstream consequences” of sex—or what have you). But this seems to us a foot-stamping response. After all, the only reason the question, “How many sexes are there?” arises in this context is because of the aforementioned disputes about how actual persons should be classified in terms of sex (for example, for legal purposes) by virtue of their diverse features. It does not help, then, to change the topic and assert that one may only use “sex” in the abstract sense employed by biologists.

The existence of people who have what are sometimes called intersex traits, DSDs, or innate variations in sex characteristics is, again, relevant to the point we are making. So, too, is the existence of transgender people—perhaps especially those who have undergone hormonal or surgical interventions to alter their biology in significant ways. We suggest that the existence of these populations creates complexity when we think about how the word “sex” can or should be used, defined, or understood (e.g., in considering its allegedly binary nature), not just in theory, but also in practice.

## Another Interpretation: Sex in Practice

Consider the following example. I am a doctor and you are my (let’s say) intersex patient. In the womb, you took a turn along one of the various pathways of sexual development that leads to a less common, but very real, destination. You have a complex constellation of traits that differs—in more or less salient ways—from biologists’ idealizations. Now, suppose it is your first time coming to my clinic and I don’t yet know you as an intersex person.

I ask, while handing you a form to fill out, “What sex are you?”

Now, let’s think.

What am I really asking here? What is the point of this question? What am I trying to find out in seeking to classify you according to sex? (see Earp, 2020 and 2021 for a similar approach to questions about gender).

In principle, I might be trying to find out any number of things. And indeed, it might be better if I had asked a more specific question about the complex set of traits you possess. However, one thing I should not be trying to find out by asking your sex (if I am doing my job properly) is, “Which of two sizes of gamete would your body hypothetically produce if it were structurally and functionally organized according to an idealized body plan type as shaped by natural selection over evolutionary time?” That definition, we submit, is neither useful nor appropriate in the given context.

Instead, I should be interested in the particular features of your body that will allow me to administer to your health effectively, while also showing you respect as a person. A box marked “sex”—with two response options—is unlikely to be enough for me to do my job well in either respect. Indeed, regardless of whether there are two, three, or even more options for sex, I will need to know more about the details of your sexed embodiment (as well as your medical history, your personal values, and so on), to attend to your needs. More generally, to better serve all my patients, I should be interested to learn about the many ways that sex can manifest in reality.

## An Objection

Of course, this is not a slam-dunk argument. In response, someone committed to a one-definition-only, evolutionary biology approach could protest that we are mixing up two different issues.

“Asking how sex is fundamentally defined,” they might say, “is not the same thing as asking how many options we should put on a medical form that inquires about an individual’s sex for certain pragmatic purposes.” It might well be appropriate to list more than two options for “sex” on such a form—this argument concedes—for example, to accommodate the experiences or needs of persons with intersex traits as well as some transgender people. But, this objection continues, the existence of such individuals does not mean that sex itself is not strictly binary.

We can imagine our interlocutor now banging their fist on the table, perhaps rounding out their case with the following: “Since, as we have established, there are only two gamete types in anisogamous species; since sex essentially refers to the means by which a given type of organism reproduces; since anisogamous reproduction requires two different body plans, defined functionally in terms of gamete production; and since humans reproduce anisogamously, there are exactly two sexes in humans. The fact that some actual human persons cannot be unambiguously categorized as belonging to one and only one of those two abstract sex categories does not entail that there are more than two sexes; it just means that some people are disordered.”

On this view, again, sex simply is the distinction between idealized body plan types that are functionally oriented around the production of a given size of gamete, of which, in human beings, there are only two. Since neither people with intersex traits nor trans people ever instantiate a body plan type that is functionally organized to produce a third (or fourth, etc.) type of gamete, their existence does not disturb the sex binary in its strictest sense, according to this objection.

We are happy to concede that what has just been described is one of the things the word “sex” can be used to pick out. It can be used to distinguish between two different idealized body plan types as explained. However, another way to use “sex”—one that is no less legitimate, in our view, given the diversity of purposes for which one might reasonably wish to use that word in human societies—is to pick out different ways that actual human beings can be differently embodied in reality. Not in terms of what gametes they would hypothetically produce if one first abstracts away from various particulars, but rather, in terms of their actual body plans: the tangible, real, instantiated bodies that diverse human beings actually have.

To insist that “sex” may only be used to describe an abstraction—an idealized model used for prediction and explanation at the species-level, based on evolutionary principles—but may not be used to describe the actual phenomenon that is purportedly being modeled as it exists in the real world (with all its manifest variation), seems to us tendentious.

In theory, sex is a binary. In reality, sex shows variety.

## The Toy Factory

Imagine a toy factory that has been explicitly set up to produce two—and only two—kinds of toy figurines. One kind of figurine is designed to have rosy skin, coral hair, and salmon-colored attire. We’ll call this a pink-type toy. The other kind of figurine is designed to have cool-toned skin, navy hair, and cobalt attire. We’ll call this a blue-type toy. All machinery and protocols were selected for the purpose of creating one or the other of these two toy-types (and nothing else). The factory is oriented toward a binary output.

Yet, actual production reveals that while most toys are clearly and unambiguously pink or blue, a consistent percentage emerges with intermediate colorations: lavender, purple, periwinkle, and plum. These “variant” toys are not defective *qua* figurines—they are whole, working products of the factory’s actual processes. They exist. They are real. And they have their own color. A color that is not simply pink or blue.

So, how many different colors of toy are there?

To be sure, our factory’s core architecture relies on a binary logic. It aims to produce, and mostly produces, the expected pink and blue toys. However, inherent production variability across numerous parameters—fluctuations in dye-mixing, timing, temperature, and so on—results in a consistent stream of intermediate-colored toys: somewhere between pink and blue.

A person approaching the factory with a mentality fixed solely on verifying design adherence might characterize these toys as, say, the unfortunate outcome of “dye-mixing variability.” They might dismiss them as rounding errors, insisting that the very basis and origin of color differentiation among toy figurines is the exclusively pink versus blue orientation of the factory's toy-making machinery.

But if the question is “What colors of toy does this factory actually produce?”, then purple, lavender, and periwinkle toys must necessarily be included for completeness and accuracy.

By contrast, claiming that there are only two colors of toy, while pointing to the factory’s design plans, would be obtuse. It would also be empirically untenable. And it would miss the point of the question. Claiming that “color” just means (or can only legitimately be used to pick out) “the distinction between different types of factory equipment according to which toy-type each was designed to produce,” while noting that there are exactly two kinds of such equipment—with no “intermediate” machines—would also be unhelpful, in our view.

Now, imagine these toys come to life. They form a society. The pink and blue majority design institutions around their color identities: segregated public spaces, different sports leagues, color-coded legal identification.

Purplish toys struggle to fit in.

Which bathrooms should a lavender toy use? Which sports league suits a periwinkle toy?

Forms for collecting toy demographics offer only two choices—pink or blue. Medicine develops separate protocols based on assumed color-correlated physiology, forcing purplish toys into ill-fitting care pathways. Doctors even start to subject purple toys to harsh dye treatments, without their consent, to try to “fix” their abnormality.

The scientists in toy society—almost all of whom are, themselves, either pink or blue—are primarily interested in understanding how toys come to exist, and why they tend to have the features that they have. One day, they discover their own factory’s blueprints and adopt a strict binary definition of color.

Toy doctors, in turn—who are also, overwhelmingly, either pink or blue—point to the scientists’ definition to justify their practice of forced re-dying. Purplish toys, they explain, are “supposed” to be either pink-type or blue-type; intermediate toys should therefore be corrected (it’s also in their best interests to be made to conform, the doctors add, when some toys start to ask questions).

When a lavender toy asks, “What color am I?”, it might have different aims in mind. It might, for example, be seeking practical information about access to certain services. It might want to know where it belongs; if it has value, etc. Indeed, it might want to know all kinds of things. But “What color would I hypothetically be if the dye-mixing process at the factory had adhered to design parameters?” is unlikely to be their main point of interest.

The truth is, their color is lavender.

When someone asks the question, “How many colors of toy are there?”, we should pay attention to who is doing the asking. Is it the pink or blue majority? Evolutionary biologists? Politicians? Whose perspective are we giving weight to?

## Completing the Analogy

Let’s bring this back to sex. If we insist on a definition according to which human sex is fundamentally binary, male or female, because this accords with evolution’s “design” for anisogamous reproduction, intersex variations are then likely to be classed as deviations—“disorders of sex development”—rather than seen as challenges to the binary. This would be like the factory owner declaring a purple toy a “defective pink” rather than seeing it as having a color of its own. Such insistence, we suggest, is not an objective requirement of science or scientific thinking. Instead, it is a political decision—one that, moreover, “stipulates away” the very point of contention. In other words, defining sex *a priori* (only) in terms of its underlying evolutionary logic makes binary conclusions tautological; observed variations are said not to be constitutive of sex, but only “downstream” implications or effects of sex, or the result of something having gone awry.

But why should that be the only admissible use of the term?

Our argument is that “sex” can mean more than one thing, and it depends on what question we seek to answer. If the question is something analogous to “What is the design plan for color types of toys according to factory blueprints?”, a strict binary definition may be appropriate. If it is something more like “What is the full spectrum of colors actually produced by the factory?”, we must look beyond pink and blue.

Of course, acknowledging the existence of purple toys does not negate the fact that most of the toys leaving the factory—possibly, the vast majority—are either pink or blue. Likewise, there are males and females in the world, and most people can indeed be parsimoniously classified as one or the other (at least in terms of innate bodily traits; we are not here referring to gender). A comprehensive understanding of the nature of both toykind and humankind involves holding more than one truth in mind: there are both common patterns rooted in (evolutionary) design principles and real variation extending beyond these patterns.

## Final Thoughts

The “toy factory” analogy has many limitations. It does not correspond to every element of sex or capture every aspect of associated debates. However, in providing this analogy, we have tried to show that a coherent response to questions around defining sex—and counting sexes—depends on one’s aims and purposes. Are we trying to understand evolutionary design principles or make sense of people’s lived realities? Are we talking about blueprints or buildings? Seemingly objective scientific disagreements often mask deeper conflicts about priorities, intentions, or values.

In toy society, insisting that “color” is definitionally binary (in its strict sense) by referring to factory blueprints obscures the diversity of actual colors observed in factory outputs. Analogously, defining sex exclusively in terms of idealized body plans forecloses the use of complementary, and no less scientific, definitions that are needed to describe actual bodies in all their observable complexity. By recognizing both variation and pattern and explicitly acknowledging the values driving our definitional frameworks, we can foster a more comprehensive, accurate, and humane understanding of sex as it exists in our world.

## Footnotes

1 In other words, even the smallest of the large-type gametes (ova or eggs) is always many times larger than the biggest of the small-type gametes (sperm). In humans, for instance, eggs are thousands of times larger than sperm by volume (Alberts et al., 2022; Bastawros, 2023; see also Fusco & Minelli, 2019). Thus, even accounting for variation in size within each gamete type, there are likely no sperm that are even approximately as big as any egg within a single species.
